# Brain dynamics predictive of response to psilocybin for treatment-resistant depression

**DOI:** 10.1093/braincomms/fcae049

**Published:** 2024-02-15

**Authors:** Jakub Vohryzek, Joana Cabral, Louis-David Lord, Henrique M Fernandes, Leor Roseman, David J Nutt, Robin L Carhart-Harris, Gustavo Deco, Morten L Kringelbach

**Affiliations:** Department of Psychiatry, University of Oxford, Oxford, UK; Center for Music in the Brain, Aarhus University, Aarhus, Denmark; Center for Brain and Cognition, Computational Neuroscience Group, Department of Information and Communication Technologies, Universitat Pompeu Fabra, Barcelona, Spain; Department of Psychiatry, University of Oxford, Oxford, UK; Center for Music in the Brain, Aarhus University, Aarhus, Denmark; Life and Health Sciences Research Institute (ICVS), School of Medicine, University of Minho, Braga, Portugal; ICVS/3B’s—PT Government Associate Laboratory, Braga/Guimarães, University of Minho, Portugal; Department of Psychiatry, University of Oxford, Oxford, UK; Center for Music in the Brain, Aarhus University, Aarhus, Denmark; Department of Psychiatry, University of Oxford, Oxford, UK; Center for Music in the Brain, Aarhus University, Aarhus, Denmark; Centre for Psychedelic Research, Department of Brain Sciences, Imperial College London, London, UK; Centre for Psychedelic Research, Department of Brain Sciences, Imperial College London, London, UK; Centre for Psychedelic Research, Department of Brain Sciences, Imperial College London, London, UK; Psychedelics Division, Neuroscape, Department of Neurology, University of California San Francisco, San Francisco, CA, USA; Center for Brain and Cognition, Computational Neuroscience Group, Department of Information and Communication Technologies, Universitat Pompeu Fabra, Barcelona, Spain; Institució Catalana de la Recerca i Estudis Avançats (ICREA), Barcelona, Spain; Department of Neuropsychology, Max Planck Institute for Human Cognitive and Brain Sciences, Leipzig, Germany; School of Psychological Sciences, Monash University, Melbourne, Australia; Department of Psychiatry, University of Oxford, Oxford, UK; Center for Music in the Brain, Aarhus University, Aarhus, Denmark; Life and Health Sciences Research Institute (ICVS), School of Medicine, University of Minho, Braga, Portugal

**Keywords:** large-scale brain modelling, psilocybin treatment, depression

## Abstract

Psilocybin therapy for depression has started to show promise, yet the underlying causal mechanisms are not currently known. Here, we leveraged the differential outcome in responders and non-responders to psilocybin (10 and 25 mg, 7 days apart) therapy for depression—to gain new insights into regions and networks implicated in the restoration of healthy brain dynamics. We used large-scale brain modelling to fit the spatiotemporal brain dynamics at rest in both responders and non-responders before treatment. Dynamic sensitivity analysis of systematic perturbation of these models enabled us to identify specific brain regions implicated in a transition from a depressive brain state to a healthy one. Binarizing the sample into treatment responders (>50% reduction in depressive symptoms) versus non-responders enabled us to identify a subset of regions implicated in this change. Interestingly, these regions correlate with *in vivo* density maps of serotonin receptors 5-hydroxytryptamine 2a and 5-hydroxytryptamine 1a, which psilocin, the active metabolite of psilocybin, has an appreciable affinity for, and where it acts as a full-to-partial agonist. Serotonergic transmission has long been associated with depression, and our findings provide causal mechanistic evidence for the role of brain regions in the recovery from depression via psilocybin.

## Teaser

Vohryzek *et al*. investigated the effects of psilocybin therapy on depression. They employed brain modelling and uncovered crucial regions involved in restoring a healthy brain from a depressive brain state. Interestingly, these regions aligned with serotonin receptor density maps, linking psilocybin’s effects to serotonergic pathways implicated in depression recovery.

## Graphical Abstract

**Graphical Abstract:**
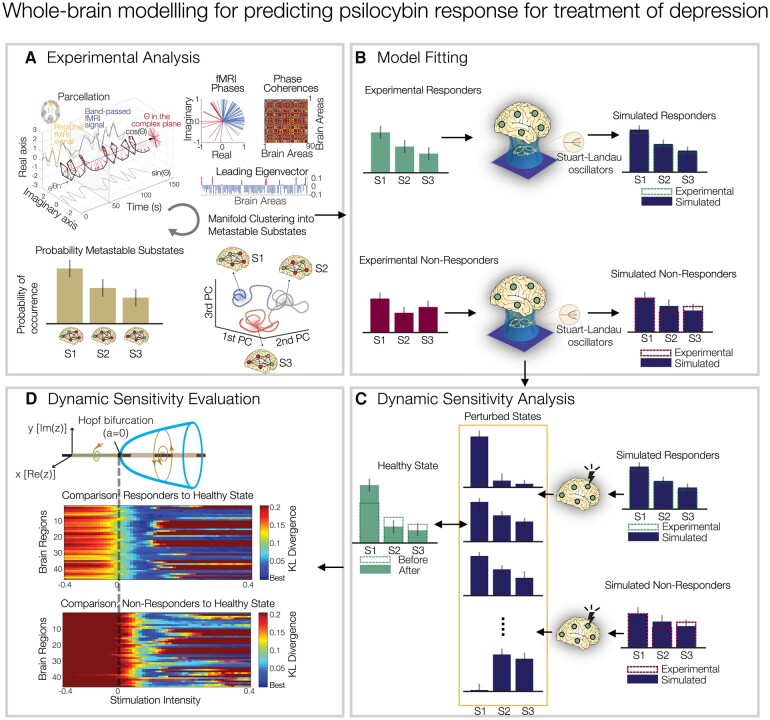

See Çatal and Northoff (https://doi.org/10.1093/braincomms/fcae067) for a scientific commentary on this article.

## Introduction

Behavioural differences between healthy and depressed individuals can sometimes be conspicuous, but identifying causal contributions from brain dynamics is more challenging. Discrete global brain states, such as those that pertain to sleep, healthy waking consciousness and the psychedelic state, have their own characteristic spatio-temporal dynamics, involving large-scale spatial communities temporally evolving in transient arrangements.^1-4^ With recent advancements in non-invasive neuroimaging techniques, it has become possible to describe complex spatio-temporal dynamics in terms of their spatial and temporal information. Still, one of the challenges for systems neuroscience is to understand what the most appropriate description of such dynamics is and how transition from one state to another is made possible.

A common method for characterizing global brain function involves assessing how activity is temporally correlated across spatially separate brain areas over an entire recording period, defining static and state-specific ‘functional connectomes’.^5-7^ However, the last decade has brought clear evidence that finer-grained, more dynamic analysis of brain states can deepen our understanding of their properties and relationship to behavioural states.^8-10^ There is a growing taxonomy of approaches to characterize the dynamics of functional interactions,^11-13^ from data-driven heuristic clustering methods across time,^8-10,14^ dynamical systems-informed phase-locking approaches,^15-17^ hidden Markov models^18,19^ to spatio-temporal networks.^20,21^

From a clinical perspective, major depressive disorder (MDD) lends itself ideal for the dynamic analysis as its symptomatology is often defined by ruminative states with self-critical and inflexible periods of thinking. Such behaviour has been linked to the abnormal spatio-temporal organization of resting state activity.^22^ Converging findings have found the overactivation of default mode network (DMN) and hypoconnectivity in the control executive network in MDD.^23-25^ This is consistent with the triple-network model whereby depressive states promote the inward and self-referential experience at the expense of the interactions and attention towards the external environment.^24,26^ In addition, the MDD brain state has been found to reflect greater synchronization and temporal stability of overall brain activity compared to healthy participants.^27^

Efforts and methods are advancing for understanding response to neuropharmacological interventions for depression. Understanding the therapeutic actions of interventions promise not only to shed light onto the mechanistic relationship between various brain states implicated in health and pathology but also to provide inspiration for the development of new, improved interventions. However, there are considerable practical and ethical challenges for answering mechanistic questions in humans, elevating the use of animal models (with sometimes questionable translational validity) or small clinically relevant populations.^28,29^ One potential advance in this direction is the use of large-scale brain modelling—as a tool for understanding pathological changes in neuropsychiatric disorders, and, potentially, for clinical diagnosis and prediction.^4^ We are mindful, however, that the predictive power of any model depends on how well it can describe and predict experimental data to which it is fitted.^30-33^

The present paper focuses on large-scale brain network models where region-specific stimulation or excitation can be tested *in silico* and used to describe and predict empirical-informed target states^1^—such as the global brain state found in people with intractable depression. These models link regional dynamics with the neuroanatomical structure of the brain to describe the spatio-temporal activity of functional data.^34^ This approach bypasses the ethical constrains of human or non-human animal experimental settings, enabling many types of stimulation to be tested, in order to evaluate the role of regions and their excitation on transit between states—with relevance to empirical phenomena of interest. The validity of this strategy has previously been demonstrated in the context of sleep and awake states.^1^

Here, we build on this notion of dynamic sensitivity analysis to gain insight into the response to psilocybin therapy for treatment-resistant depression. We define brain states in terms of spatial subdivisions and their probability of occurrence across time, characterized as probabilistic metastable substates (PMSs). These recurrent metastable substates can be characterized by their probability of occurrence. Beyond the quantitative description of brain states, we wish to understand which brain regions play a prominent role in the recovery from depression after treatment with psilocybin.^35^

Using data from a trial of psilocybin therapy for treatment-resistant depression, the sample was binarized into ‘responders’ and ‘non-responders’ to psilocybin therapy. Functional MRI (fMRI) data were collected before and 1 day after the second of two psilocybin therapy dosing sessions. Using parameters from the empirical data, modelled brain states—and stimulation parameters therein, could then be used to predict treatment response, defined as a >50% reduction in symptom severity from baseline—determined at a key 5-week post-treatment end-point.^36^

Psychedelic medicine has shown a promising avenue for treating depression.^37^ For depression treatment, one current hypothesis is that, via a psychedelic drug × psychological intervention combination, there is an increase in global brain flexibility, translating into a window of opportunity for breaking free of negative cognitive biases and associated ruminations.^38^ Indeed, the current research on the acute effects of psychedelic drugs suggests an increase in the repertoire of brain activity substates.^39-41^ From a neuropharmacological perspective, psilocybin—an active compound in magic mushrooms—binds with high affinity to the serotonergic 5-hydrotryptamine 2a (5HT_2a_) receptors but other serotonergic receptors are also implicated.^42,43^ Psilocybin acts as an agonist resulting in higher neuronal excitability, modulating the excitatory–inhibitory balance (in favour of excitation) in the cortical brain regions with more 5HT_2a_ receptors.^44^ Recently, a large-scale brain computational study focusing on the human brain action of lysergic acid diethylamide—which has a similar pharmacology to psilocybin/psilocin—demonstrated, for the first time, the causal impact of 5HT_2a_ agonism-induced excitation on global brain dynamics.^45^

Here, in empirical fMRI data, we identified recurrent brain substates in terms of the PMS space across all the subjects in the pre- and post-treatment conditions. Furthermore, we use a computational large-scale brain model—where each brain area is represented by a Hopf bifurcation model^46^—to simulate the brain network dynamics in patients before the treatment. Through dynamic sensitivity analysis, we were able to identify brain regions responsible for treatment response at a key 5-week end-point.^1,47^  *A priori*, we hypothesized that regions permitting transition to a healthy brain state (as predicted by the 5-week end-point) would relate to the distribution of the 5HT_2a_ and 5-hydrotryptamine 1a (5HT_1a_) receptors in the human brain, as determined by prior *in vivo* PET mapping.^48^

## Materials and methods

### Experimental data

#### Functional MRI

We carried out the analysis on previously published data set of patients with treatment-resistant depression undergoing treatment with psilocybin at Imperial College London.^36^ In brief, we investigated 15 patients (without excessive movement and other artefacts from the original 19 patients) who were diagnosed with treatment-resistant major depression. The MRI scanning sessions were completed pre-treatment with psilocybin and 1-day post-treatment with the treatment consisting of two oral doses of psilocybin (10 and 25 mg, 7 days apart). The patients were split into responders and non-responders to the treatment based on the Quick Inventory of Depressive Symptomatology (QIDS) at 5 weeks post-treatment with 6 out of the 15 patients meeting criteria for response.^49^

#### Structural connectivity

In this study, white matter (structural) connectivity of 90 automated anatomical atlas brain areas from a previously obtained data set was used for the large-scale brain network model. In brief, the group consisted of 16 healthy young adults (5 females, mean SD age: 24.7 ± 2.54). Diffusion tensor imaging was applied following the methodology described in Cabral *et al.*^50^ Undirected structural connectivity (SC) $C_{np}$ was obtained were ***n*** and ***p*** are brain areas and the connectivity weights are defined as the proportion of sampled fibres in all voxels in region ***n*** that reach any voxel in region ***p***. Finally, the individual structural connectomes were averaged across the 16 subjects to obtain a group-based template.

#### Probabilistic metastable substates

Firstly, we calculated the instantaneous phased relationship between individual brain regions by expressing the demeaned regional fMRI signal x(t) as an analytical signal, i.e. in terms of its time-varying phase θ(t) and amplitude A(t) as x(t)=A(t)*cos(θ(t)).^51^ We excluded the first and last three timepoints to account for the boundary artefacts introduced by the Hilbert transform. Hence, for every timepoint *t* and pair of brain regions *n* and *m*, we obtain the phase coherence matrix dPC as follows:


(1) dPC(n,m,t)=cos(θ(n,t)−θ(m,t)).

By decomposing the signal in this way, we can look at when the brain regions *n* and *m* are aligned with similar angles, cos(0)=1, orthogonal to each other, cos(π/2)=0 , and anti-aligned, cos(π)=−1. As the phase coherence is a measure of undirected connectivity, the phase coherence matrix dPC is symmetric and all the meaningful information is captured in the upper triangular matrix.

For further analysis, we used only the *1xN* leading eigenvector $V_{1}(t)$ of the dPC matrix as described in the Leading Eigenvector Dynamics Analysis (LEiDA).^15^ In detail, at every timepoint *t* of the dPC(t), we performed the eigendecomposition taking the first (most dominant) eigenvector to describe the dPC(t) pattern. The dPC(t) is decomposed as $\mathrm{dPC}(t)=V(t)D(t)V^{-1}(t)$, where *D* is the diagonal matrix carrying the real-valued eigenvalues and $V_{1}(t)$ and $V_{1}^{-1}(t)$ are the left and right corresponding orthogonal eigenvectors, respectively. The dominant connectivity pattern can be simply reconstructed by the following matrix multiplication: $V(t)V^{-1}(t).$

The next step was introduced to find recurrent phase-locking substates in the dominant connectivity patterns varying in time. To that end, we clustered all the leading eigenvectors obtained from all the fMRI scans obtained from both responders and non-responders, thus achieving common phase-locking substates for both groups. For the clustering, we used the unsupervised *k*-means algorithm, with varying cluster numbers *k* from 2 to 10 clusters. The algorithm was run in an *n*-dimensional space where *n* = 90 (number of automated anatomical atlas brain regions) with condition × subject × timepoints number of observations. For each run, we randomly initialized the algorithm 20 times to ensure stability in the clustering. Again, by computing the matrix multiplication of the *1xN* cluster centroids $V_{c\alpha }$ as $V_{c\alpha }(t)V_{c\alpha }^{T}(t)$, we obtain the dominant connectivity pattern of each cluster. In the current analysis, we considered the cluster solution k=3 as an optimal choice between the quality measures—Dunns, Davies–Bouldin and silhouette score, and Davies (Supplementary Fig. 2), and the maximizing of the statistical significance between patient groups (*P*-values).

After calculating the phase-locking states, we defined the probability of occurrence of the individual substates by simply dividing their occurrence in each recording session by the total number of timepoints recorded (same for all recordings).

#### Large-scale brain computational model

In order to simulate the ultra-slow fluctuations in fMRI signal detected during rest, we used the Landau–Stuart oscillator canonical model, describing the transition from a noisy to an oscillatory dynamics.^52^ The so-called supercritical Hopf bifurcation model was used locally at every brain region (node) to emulate the local dynamics.^1,46^ To achieve a large-scale brain-level description, the individual Hopf models were coupled in a SC network, describing the large-scale white matter map of the human brain.^46,53^ The emerging and complex interactions in the large-scale brain network of coupled Hopf models have been shown to describe many aspects known from experimental recordings in MEG^54^ and fMRI.^1,46,47,55,56^

Formally, the normal form of the supercritical Hopf bifurcation model for a single uncoupled region of interest (*n*) in Cartesian coordinates is described by the following set of coupled equations:


(2) $$\frac{dx_{n}}{dt}=(a_{n}-x_{n}^{2}-y_{n}^{2})x_{n}-\omega_{n}y_{n}+\beta \eta_{n}(t),$$

(3) $$\frac{dy_{n}}{dt}=(a_{n}-x_{n}^{2}-y_{n}^{2})y_{n}+\omega_{n}x_{n}+\beta \eta_{n}(t),$$

with $\beta \eta_{n}(t)$ being the Gaussian noise with SD of β=0.02. The bifurcation parameter a positions the system at the supercritical bifurcation point when a=0, noise activity governed by $\beta \eta_{n}(t)$ in regime when a<0 and stable limit cycle with oscillatory behaviour of frequency defined by $f_{n}=\omega_{n}/2\pi$ when a>0. The values of the intrinsic frequency *ω* were calculated from the experimental fMRI signals in the 0.04−0.07Hz band by taking the peak frequency of the Gaussian-smoothed power spectrum of each brain area.

To describe the coupled large-scale brain computational model, we introduced the coupling term (modelled as the common difference coupling, i.e. describing the linear term of a general coupling function) between the individual nodes weighted by the corresponding values of the SC matrix. To be noted, we do not consider the next non-linear coupling term following Taylor expansion of the full coupling, in case the linear coupling is non-existent.^57,58^ Equations (2) and (3) can be hence expanded as follows:


(4) $$\begin{array}{rl} \frac{dx_{n}}{dt}=\; & \left(a_{n}-x_{n}^{2}-y_{n}^{2})x_{n}-\omega_{n}y_{n}+G\overset{N}{\underset{\;p=1}{\sum }}\;C_{np}(x_{p}-x_{n}\right)\\ & +\beta \eta_{n}(t), \end{array}$$

(5) $$\begin{array}{rl} \frac{dy_{n}}{dt}=\; & \left(a_{n}-x_{n}^{2}-y_{n}^{2})y_{n}+\omega_{n}x_{n}+G\overset{N}{\underset{\;p=1}{\sum }}\;C_{np}(y_{p}-y_{n}\right)\\ & +\beta \eta_{n}(t), \end{array}$$

where $C_{np}$ is the SC weight between node *n* and *p*, and *G* is the global coupling weight with equal contribution between all the nodal pairs. The SC matrix was rescaled to have the mean value ⟨C⟩=0.2 in order to be consistent with previous literature’s range of parameters.^1,46^ The simulated signal is described by the $x_{n}$ equation for every node *n*. The variables *G* and *a* are the control parameters used for the model fitting to the experimental data and the stimulation protocol, respectively.^1,46^

#### Objective function

In order to validate the simulated signal, different realizations of the experimental data can be used.^30^ The most standard approach is comparison of the simulated data with grand-averaged static functional connectivity as computed by the Pearson correlation^34,59^ or metastability defined as the SD of the Kuramoto order parameter (Supplementary Fig. 4—Metastability). To account for the temporally varying nature of the blood oxygen level–dependent signal, recent literature has focused on the comparison between the simulated and empirical functional connectivity dynamics (FCD) spectrums (quantified by the Kolmogorov–Smirnov distance), i.e. the distributions of the cosine distance between the consecutive timepoints as described by the leading eigenvector^45,46^ (Supplementary Fig. 4—Functional Connectivity Dynamics). As alluded to in the previous section, the fMRI signals organize into spatially meaningful phase-locking states. Here, we compare the simulated data to the probabilities of occurrence of the phase-locking states found in the experimental recordings.^1^ We used the symmetrized Kullback–Leibler divergence (KL divergence) of the simulated and empirical probabilities of occurrence as follows:


(6) $$KL(P_{\mathrm{emp}},P_{\mathrm{sim}})=0.5\left(\left(\underset{i}{\sum }\;P_{\mathrm{emp}}(i)\ln\frac{P_{\mathrm{emp}}(i)}{P_{\mathrm{sim}}(i)}\right)+\left(\underset{i}{\sum }\;P_{\mathrm{sim}}(i)\ln\frac{P_{\mathrm{sim}}(i)}{P_{\mathrm{emp}}(i)}\right)\right),$$

with $P_{\mathrm{emp}}$ and $P_{\mathrm{sim}}$ being the empirical and simulated probabilities of occurrence of the same phase-locking states, respectively.

#### Statistical analysis

We use non-parametric permutation tests for the experimental analysis with 1000 permutations. For the receptor analysis, we used standard Pearson’s correlation. Both tests were implemented in MATLAB.

## Results

**Figure 1:**
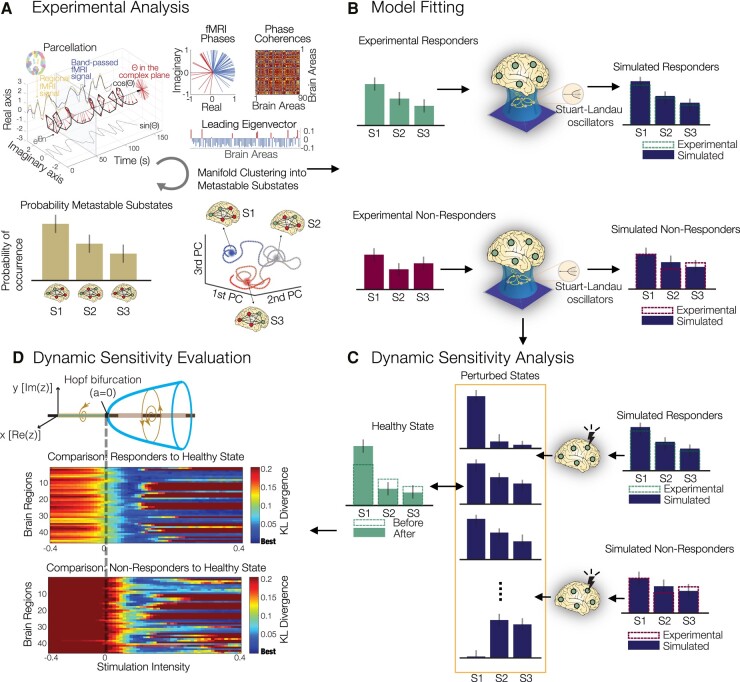
**Study overview.** (**A**) Experimental analysis. PMSs were computed for each condition using LEiDA. Regional fMRI time series were first converted to analytical signal, followed by computation of the leading eigenvector of the phase coherence matrix at every timepoint. An unsupervised *k*-means algorithm was deployed to cluster the eigenvectors into a three-substate solution. The PMS is defined as the probability distribution of substates, obtained for each individual scan and averaged within each condition. S1, substate 1; S2, substate 2; S3, substate 3. (**B**) Model fitting. Large-scale brain model parameters were optimized to fit the PMS before treatment separately for responders and non-responders. (**C**) Dynamic sensitivity analysis. *In silico* bilateral perturbations were performed to find the optimal protocol to transition to the PMS characteristic of a healthy brain state [described by responders’ (as predicted by the 5-week QIDS end-point) 1 day post-treatment brains]. (**D**) Dynamic sensitivity evaluation. Perturbations are applied separately in each pair of bilateral brain regions by varying the intensity of oscillations as defined by the bifurcation parameter *a*.

In summary, a quantitative characterization of the spatio-temporal dynamics recorded with fMRI was obtained using LEiDA, resulting in the definition of PMSs (Fig. 1A), whose probability of occurrence was compared across conditions (i.e. within-subject design—therefore, before versus after treatment). We then constructed two large-scale brain models representative of the pre-treatment brains to psilocybin therapy. This was done by fitting their PMS descriptions to those obtained from the experimental data (Fig. 1B). Finally, a dynamic sensitivity analysis was implemented to both responder and non-responder pre-treatment models to identify the brain regions that permit a transition to the healthy PMS [described by responders’ (as predicted by the 5-week end-point) 1-day post-treatment brains; Fig. 1C and D].

**Figure 2:**
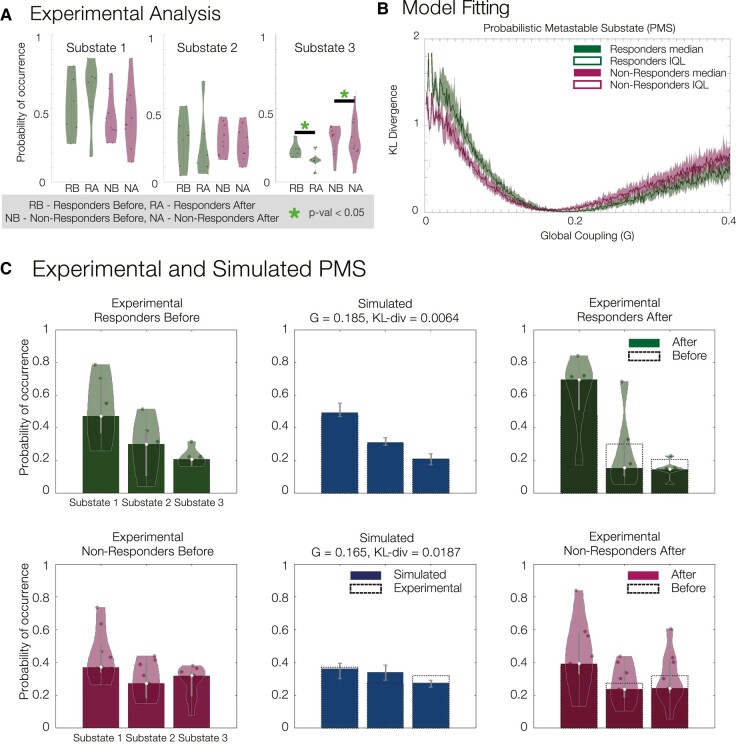
**(A) Experimental analysis.** Probability of occurrence (or fractional occupancy) of each metastable substate detected with LEiDA for the three-substate clustering solution. Significant differences were observed in substate 3 between responders before and after treatment (*P* = 0.0258, signed rank-sum permutation test, star for significance, number of responders 6 and number of non-responders 9, individual subjects plotted for the experimental analysis, **A**); responders and non-responders after treatment (*P* = 0.0141, rank-sum permutation test, star for significance, number of responders 6 and number of non-responders 9, individual subjects plotted for the experimental analysis, **A**); and no significant differences were found between responders and non-responders before treatment. (**B**) Model fitting of the responder and non-responder models as a function of the global coupling parameter *G*, with optimal fits at *G* = 0.185 (KL divergence = 0.0064) and *G* = 0.165 (KL divergence = 0.0187), respectively. We report the median across subjects in each group with interquartile range (IQL). (**C**) Experimental and simulated PMS. Experimental PMS for responders and non-responders before treatment (left), their simulated counterparts at optimal *G* (middle) and experimental PMS for responders and non-responders after treatment (right).

As described in the ‘Materials and methods’ section, we computed the PMS pre- and post-treatment with psilocybin (where ‘post’ = 1 day post psilocybin dosing session two), for both responders and non-responders (determined 5 weeks hence). Here, we focused on a three-substate solution—the lowest *k*-level with statistically significant differences between the two groups as well as optimal quality measures across clustering solutions (Supplementary Fig. 2). When contrasting responders versus non-responders, the occurrence of substate 3 was significantly different pre- versus post-treatment (*P* = 0.0258, signed rank-sum test), as well as in the post-treatment data alone (*P* = 0.0141, rank-sum test; Fig. 2A). Furthermore, we also computed the global brain connectivity, metastability and FCD measures (Supplementary Fig. 1). These results clearly indicated the necessity of considering both spatial and temporal dimensions to differentiate between conditions as global brain connectivity, synchrony and metastability show non-significant results. Conversely, the FCD measure showed significant differences in the temporal similarities of spatial patterns between pre- and post-treatment responders (*P* = 0.0163, signed rank-sum permutation test) and pre- and post-treatment non-responders with post-treatment responders, respectively (*P* = 0.0183 and *P* = 0.0273, rank-sum permutation test), further supporting the use of spatio-temporal measures to capture the alterations in large-scale brain dynamics across conditions.

To obtain large-scale brain computational models representative of the two groups of patients (responders and non-responders before treatment), we first defined a generalized brain network model, where each of 90 cortical and subcortical brain regions (defined using automated anatomical labelling^60^) was described by a Stuart-Landau oscillator (see the ‘Materials and methods’ section), and regions were coupled according to realistic SC obtained from diffusion MRI.

To adjust the model to each group of patients, first, the intrinsic frequency of each brain region was set to the peak frequency in fMRI signals averaged across patients in the same group (Supplementary Fig. 3). Subsequently, the global coupling parameter, *G*, was tuned to optimize each model to its appropriate working point. This was achieved by minimizing the divergence between the experimental and simulated PMS spaces (see Fig. 2B). In Supplementary Fig. 4, we report optimization curves for other observables such as the static FC, metastability and FCD. For the responders and non-responders before treatment, we found *G* = 0.185 (KL divergence = 0.0064) and *G* = 0.165 (KL divergence = 0.0187), respectively, to minimize the difference. Figure 2C shows, on the left, the experimental results for both groups before treatment; in the middle, the optimal simulated fits for both groups; and on the right, the experimental results after treatment (with the results of responders after treatment serving as the target PMS for rebalancing). These findings clearly demonstrate the large-scale model’s ability to fit the spatio-temporal dynamics, as described by the PMS space, of the studied groups of responders and non-responders before the treatment.

**Figure 3:**
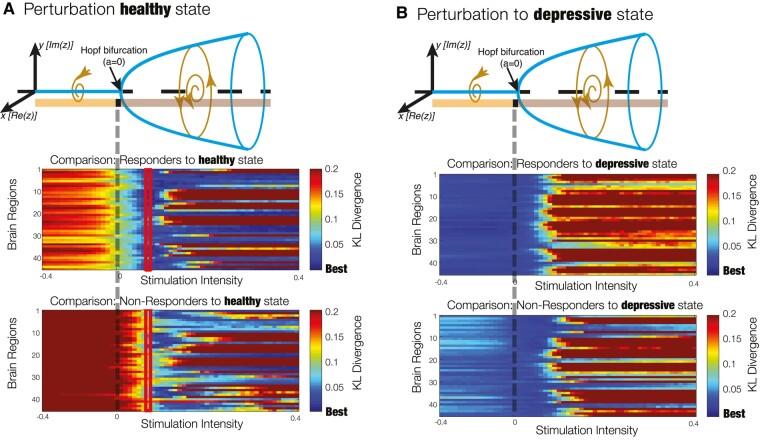
**Evaluation of dynamic sensitivity analysis.** (**A**) Perturbation to induce a transition to a healthy state. Each homological pair of brain regions was perturbed by varying the bifurcation parameter **a**, which modulates the intrinsic oscillatory behaviour of the dynamical units. The more **a** is positive, the larger the amplitude of intrinsic oscillations, whereas for negative **a**, the units decay to a fixed point equilibrium and the local dynamics is dominated by noise. The performance of the perturbations is evaluated by computing the KL divergence between the simulated PMS and the empirical PMS from patients who recovered after treatment with psilocybin. Optimal intensity of **a** = 0.07 was achieved for the responder group (highlighted rectangles). (**B**) Perturbation to induce a transition to a depressive state. A transition to the depressive state showed worse or no effect at varying values of the bifurcation parameter **a**. This is expected since the models were adjusted to patients in the depressive state before treatment.

Subsequently, we considered dynamic sensitivity analysis to determine the optimal perturbation strategies to rebalance the PMS distribution to the healthy state (as defined by the PMS space of responders after 1 day after treatment). Figure 3 illustrates the dynamic sensitivity analysis, whereby the bifurcation parameter *a* is used to change the nodal dynamics in terms of its response to added noise, ranging from a more noise-driven regime (the more **a** is negative) to an oscillatory regime (with larger amplitude, the more **a** is positive). We focused on homological nodal perturbation of the large-scale brain model, meaning that bilateral regions were perturbed equally, resulting in 45 pairs of regions perturbed at gradually varying values of *a*. Figure 3A shows the dynamic sensitivity analysis of driving a transition to the healthy state for models of both responders and non-responders before treatment. Again, an average of the KL divergence between either the perturbed pre-treatment responder or non-responder models and the healthy PMS space was shown. In the noise-driven regime (*a* < 0), a deterioration of the fit was observed for both groups, while in the oscillatory regime (*a* > 0), an initial improvement across all 45 runs was depicted, before subsequent deterioration away from the optimal fit for both groups. Conversely, when replacing the target healthy state by the depressive state (i.e. by comparing with the average PMS in non-responders after treatment), we found that the KL divergence was minimal without perturbation (i.e. keeping *a* = 0), showing a worse fit for both groups when brain areas became more oscillatory and no effect of the noisy perturbation (Fig. 3B). This analysis demonstrates the optimal response for the amelioration of brain dynamics towards the optimal brain dynamics, as described by the PMS space of post-treatment responders, to be in the oscillation-driven regime (*a* > 0) for both responders and non-responders.

**Figure 4:**
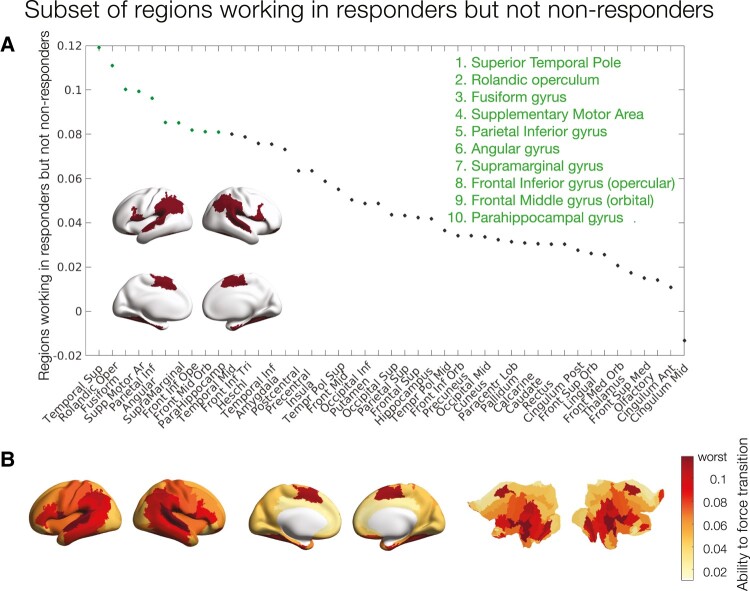
**Subset of regions working in responders but not in non-responders.** (**A**) Rank-ordered absolute difference of KL divergence between perturbations of the responder and non-responder models before treatment at a stimulation intensity of **a** = 0.07. Inset brain rendering of the 10 brain regions with the highest difference: the temporal superior pole, rolandic operculum, fusiform gyrus, supplementary motor area, parietal inferior gyrus, angular gyrus, supramarginal gyrus, frontal inferior gyrus (opercular), frontal middle gyrus (orbital) and parahippocampal gyrus. (**B**) Cortical rendering and flat maps showing the distribution of all KL divergence differences.

To evaluate which regions permitted transition to a healthy state, we first defined the optimal perturbation strength as the minimum of the averaged KL divergence (across the 45 runs) of the responder group to the treatment. This stimulation intensity was found at *a* = 0.07. Then, we inspected the difference between the responders and non-responders at that given value of *a* to assess what nodal perturbations were permitting the transition to the healthy state in responders but not in non-responders (Fig. 4).

Figure 4A shows the rank-ordered regional differences in KL divergence between perturbations of the responder and non-responder models before treatment at a stimulation intensity of *a* = 0.07. We highlighted the regions with the largest KL divergence working in responders but not non-responders to promote a transition to the healthy state. These regions are the temporal superior pole, rolandic operculum, fusiform gyrus, supplementary motor area, parietal inferior gyrus, angular gyrus, supramarginal gyrus, frontal inferior gyrus (opercular), frontal middle gyrus (orbital) and parahippocampal gyrus. Figure 4B shows the cortical rendering of these differences. In summary, these regions are implicated in transition away from ‘depressed brain’ pathology and towards the ‘healthy brain’ configurations of treatment responders.

### Correlation with serotonin receptor maps

**Figure 5:**
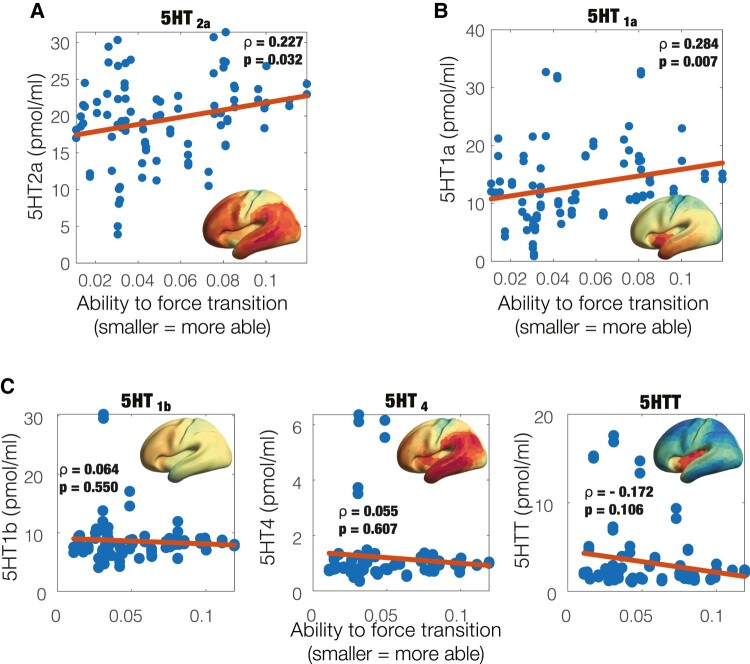
**Ability to promote a transition relates to density of specific serotonin receptors.** For each pair of homological brain regions, the ability to promote a transition is plotted against the receptor map densities of (**A**) 5-HT_2a_ (Spearman’s correlation *ρ* = 0.227, *P* = 0.032) and (**B**) 5HT_1a_ ( *ρ* = 0.284, *P* = 0.007). (**C**) In contrast, the other 5-HT receptors with non-significant results are 5-HT_2b_ ( *ρ* = 0.064, *P* = 0.055) and 5-HT_4_ ( *ρ* = 0.055, *P* = 0.607), as well as the serotonin transporter 5-HTT ( *ρ* = −0.172, *P* = 0.106).

Given the unique and known neuropharmacology of psychedelics whereby psilocybin—the active component of magic mushrooms—binds with high affinity to the serotonergic receptors (mainly the 5-HT_2a_) and in effect increases neural activity, we assessed whether the regions working in responders but not non-responders overlapped with the 5-HT density maps derived from PET imaging data previously obtained by an independent research group.^48^ Figure 5A and B show the correlation between the 5-HT_2a_ and 5-HT_1a_ receptor density maps and regional differences in KL divergence between perturbations of the responder and non-responder models before treatment at a stimulation intensity of *a* = 0.07 (same as in Fig. 4; Spearman’s *ρ* = 0.227, *P* = 0.032, and Spearman’s *ρ* = 0.284, *P* = 0.007, respectively). Figure 5C shows non-significant correlations to other 5-HT components—namely the 5-HT_2b_ (Spearman’s *ρ* = 0.064, *P* = 0.055) and 5-HT_4_ receptors (Spearman’s *ρ* = 0.055, *P* = 0.607) and the 5-HT transporter (5-HTT; Spearman’s *ρ* = −0.172, *P* = 0.106). This analysis shows that the ability to force transition towards the optimal spatio-temporal dynamics of a given region correlates with density of 5HT_2a_ and 5HT_1a_ neuroreceptors of that region.

## Discussion

In this work, we employed a large-scale brain modelling approach to evaluate potential brain change causes of response to psilocybin therapy for treatment-resistant depression. Using a novel combination of empirical data and *in silico* modelling, systematic perturbations to brain regions modelled *in silico* revealed a subset of regions implicated in transition away from ‘depressed brain’ pathology and towards the ‘healthy brain’ configurations of treatment responders. Notably, these regions matched those with the highest density of 5HT_2a_ and 5HT_1a_ neuroreceptors. This relationship is plausible given that psilocin (psilocybin’s active metabolite) is known to have an appreciable-to-high affinity for the 5-HT_1a_ and 5-HT_2a_ receptors, respectively, where it acts as an agonist, in the case of the 5-HT_2a_, potentially stimulating plasticity-related signalling cascades relevant to an antidepressant action.^61,62^

Another complementary perspective of the findings, which show the correlation between 5-HT_2a_ and 5-HT_1a_ serotonergic receptors and regions with difference in the intervention between the groups, is that of the overactivation of DMN in depression both in terms of its connectivity profile and temporal characterization.^22-24,63^ It has been shown that serotonergic raphe nuclei have direct anatomical projections to the regions of DMN and 5HT_2_ receptors expressed in DMN regions,^64^ and it is thus possible that the psychedelic action enables modulation of the DMN via serotonergic pathways in responders but not non-responders.

A summary of complex spatio-temporal dynamics, in terms of brain substates and their transitions, has drawn a lot of attention in systems neuroscience due to its utility to evaluate the impact of pharmacological and electromagnetic interventions for treating brain and behavioural disorders. Brain substates have been characterized in different ways, by minimal energy^65^ as attractor landscapes^16,34^ and, more heuristically, through sliding window analysis and unsupervised clustering.^8,9^ However, it has been challenging to find a model that is sufficiently simple and yet accurate to account for temporally and spatially complex and non-stationary data sets. Here, PMSs are built on a description of the data in terms of a probabilistic ‘cloud’ in substate space and as such can be extended to different neuroimaging modalities with higher temporal resolution, such as EEG and MEG, or potentially to more fine-grained spatial resolutions.^1,11^

Cutting-edge non-invasive brain stimulation techniques, such as transcranial magnetic stimulation and direct electrical stimulation, and new neuropsychopharmacological drugs for the treatment of psychiatric disorders have heralded a new era of localized and system-wide brain perturbations as medical interventions. For example, transcranial magnetic stimulation has been considered for the treatment of many psychiatric disorders, such as depression, schizophrenia and addiction,^66^ and classic psychedelic (drug) therapy, which, in part, targets a specific neuroreceptor (i.e. principally the 5-HT_1a_ receptor), is showing efficacy in the treatment of a broad range of conditions such as depressive, anxiety and addiction disorders.^38^ However, it seems highly likely that the mechanistic action of these interventions lies potentially well downstream of their initial action, and this action may not be straightforward.^67^ For example, how direct electrical stimulation–induced signal propagates within neuronal microcircuits remains unclear and often paradoxical^68^ and motivates theoretical neuroscience studies and *in silico* perturbation protocols. In this perspective, our study attempted to understand how the psychedelic-induced perturbation impacts regional activity that alters the brain-wide dynamics, thus potentially bridging the initial (as done experimentally) and downstream actions (as done *in silico*) of such intervention.

Beyond *in silico* perturbations, the exhaustive stimulation protocol can also be used as a dynamic sensitivity analysis tool from the complex systems perspective. Traditionally, statistical differences in measures summarizing spatio-temporal dynamics are obtained using signal detection theory. Such approaches can be enhanced by considering large-scale brain models and their structural differences between conditions, for example as described by the global coupling (*G*) parameter. Moreover, rather than describing and assessing expressions of spatio-temporal dynamics, an exhaustive protocol allows a shift of focus onto transitions to a target state, and this can be used to identify differences between groups, such as treatment responders versus non-responders, as we have done here.

Forcing transitions in large-scale brain networks has also been investigated through the prism of control network theory. In such scenarios, control strategies are deployed to navigate complex systems from a source (initial) state to a target (final) state.^69^ This approach has obtained a lot of attention due to its wide-ranging engineering applicability in technological, social and cyberphysical systems across various experimental scenarios.^70,71^ However, the conceptual understanding of controlling neuronal signals from source to target might be problematic as the brain operates in self-sustained and non-equilibrium state, and the notion of well-defined pathway between them might be ill posed.^72^ On the contrary, the approach considered in this work describes spatio-temporal dynamics in terms of PMSs and, through systematic perturbation, rebalances the spatio-temporal dynamics between two PMS spaces. Through this approach, the brain is rebalanced to its healthy working point, without specific instructions of what the relevant pathway might be.

To obtain a PMS approximation of the brain substate of interest, several methodological choices are made, which inevitably introduce several caveats. First, a regional parcellation must be chosen, which might introduce artificial spatial boundaries especially when dealing with dynamics. Secondly, the choice of clustering algorithm defines the type of substates that can be obtained. Here, we use the unsupervised learning algorithm *k*-means clustering, which has been shown to adequately represent functionally meaningful brain substates.^16^ However, alternative algorithms could be used for this purpose (e.g. *k*-medoids). Related to the experimental data, the design is an uncontrolled open-label feasibility pilot study, and as such has no placebo group and suffers from small sample size. In this context, it is relevant to consider the current study as exploratory. Hence, future replication studies are warranted to ensure robustness of the findings. Moreover, the healthy state is defined here in terms of the 1-day post-treatment scan but the responders/non-responders’ assessment is done 5 weeks after. Lastly, the large-scale brain models constructed are based on group approximations of the functional brain information and SC group template. For clinical relevance, further research will be needed to create individual-based large-scale brain models that might allow for future *in silico*–assisted personalized psychiatry.^73^

## Supplementary Material

fcae049_Supplementary_Data

## Data Availability

Raw data were generated at Imperial College London. Derived data supporting the findings of this study are available from the corresponding author upon request. The code for the analysis is available on GitHub: www.github.com/jvohryzek/psilodep_modelling.
